# Baking sunflower hulls within an aluminum envelope in a common laboratory oven yields charcoal

**DOI:** 10.1016/j.mex.2015.03.009

**Published:** 2015-04-02

**Authors:** Pablo Maximiliano Arnal

**Affiliations:** Centro de Tecnología de Recursos Minerales y Cerámica (CETMIC), CIC – CONICET La Plata, Camino Centenario y 506, CC 49 (B1897ZCA), M.B. Gonnet, Province Buenos Aires, Argentina

**Keywords:** Carbonization of biomass, Charcoal, Carbonization, Sunflower hull, Biomass, Aluminum foil

## Abstract

Charcoals have been widely used by scientist to research the removal of contaminants from water and air. One key feature of charcoal is that it keeps macropores from the parent material – though anisotropically contracted – and can even develop meso- and micropores. However, the controlled thermochemical conversion of biomass into charcoal at laboratory scale normally requires special setups which involve either vacuum or inert gas. Those setups may not be affordable in research groups or educational institutions where the research of charcoals would be highly welcome. In this work, I propose a simple and effective method to steer the thermochemical process that converts sunflower hulls (SFH) into charcoal with basic laboratory resources.

The carbonization method:

• Place SFH in an airtight aluminum envelope.
• Thermally treat SFH within the envelope in a common laboratory oven.
• Open the envelope to obtain the carbonized sunflower hulls.

## Graphical abstract

**Figure:**
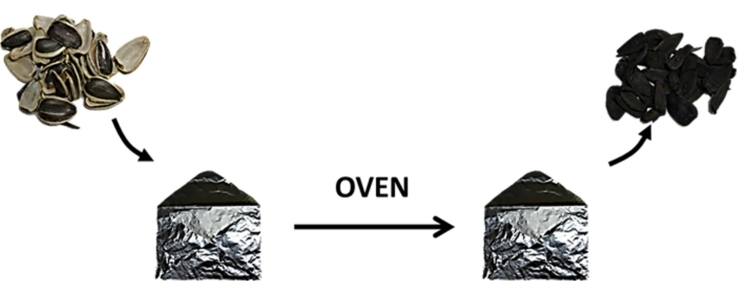

## Method details

Step 1: Place SFH in an airtight aluminum envelope.

### Materials

• Sunflower achenes (*i.e.*, seed within hull) purchased from a local food store.
• Commercially available household or aluminum foil.

The thermochemical conversion of biomass into charcoal basically consists in a process where a solid product heated in either inert atmosphere or vacuum chemically transforms into gaseous, liquid and solid products [1]. The solid becomes richer in C than SFH, while gaseous and liquid products become rich in the other main elements usually present in biomass (*i.e.*, O, H and N). The complexity of the carbonization becomes even more evident when considering that all three products may react among themselves. At the end of the thermochemical process forms a solid product (*i.e.*, charcoal), which has C as its major constituents, O as its second most abundant constituent and also somewhat less H and N. Furthermore, charcoal usually keeps a structure of macropores (>50 nm) similar to that previously present in the parent material though anisotropically shrunk and the charcoal can even develop meso (2–50 nm) and micropores (<2 nm) by suitable treatments.

However, charcoal successfully forms if the chemical reactor where the thermochemical process occurs precludes molecular oxygen (*i.e.*, O_2_) from the surface of the solid product. If oxygen would reach the solid product, then combustion would be triggered with the consequent conversion of the solid product into gaseous substances (*i.e.*, H_2_O and CO_2_ in complete combustion).

One way to preclude O_2_ (air) from the carbonization process consists in placing the biomass in an airtight envelope made with aluminum foil. Aluminum foil is very malleable and can be easily deformed without loosing its ability to block gases and water. Furthermore, it is a good thermal conductor (235 W m^−1^ K^−1^), melts at 660 °C, and is thinner than 200 μm [2], [3].

### Procedure

SFH can be simply obtained from commercially available achenes:

• Press sunflower achenes one-by-one with tongue-and-groove pliers by hand along their major axis until they crack.
• Peel cracked achenes by hand.
• Discard seeds while collecting multi-layered (pericarps or) hulls.

Once you have SFH, place them in an airtight, two-fold envelope made with aluminum foil:

• Prepare the aluminum foil:

1 Cut two squares (ca. 28 cm × 28 cm) of aluminum foil from the commercial roll.
2 Lay one of those metallic foils on a bigger sheet of paper placed on a table.

Note: the paper prevents the aluminum foil from being cut by small, solid objects laying on the table.

3 Fold the foil by joining two opposite sides while leaving the matter surface inside and the shinier surface outside.
4 Press the fold with a finger and moved it from one end to the other of the fold.
5 Unfold the aluminum foil.

• Put 1 g of SFH at the center and fold again, so that SFH remain within the rectangle formed by the bent foil.
• Seal the longest, open side:

1 Mark a parallel line with a ruler at *ca*. 5 mm from the border from the largest side of the rectangle, where the extremes of the foil joined.
2 Bend the 5-mm-high rectangle 180°.
3 Press with one finger from one extreme to the other.
4 Repeat steps 1–3 twice while taking care not to cut the aluminum foil with the SFH from the inside.
5 Press the foil by hand from the outside to remove as much air as possible from the space around the SFH.

• Seal the two shortest sides by following the same procedure described above for the longest, open side.
• Wrap the envelope containing SFH with the second squared piece of aluminum foil sealing the longest and minor open sides as just described.

Step 2: Thermally treat the SFH within the envelope in a common laboratory oven.

### Materials

• SFH within an airtight envelope of aluminum.
• Laboratory oven.
• Crucible tong.
• Isolating gloves.
• Safety goggles.

Once SFH are within the airtight envelope, the thermochemical process can go on. An increase of the temperature in a common laboratory oven triggers the thermochemical process. With this type of oven, a heating process with a linear increase of temperature followed by an isothermal step can be usually performed. Some ovens even allow performing several of these heating-and-isothermal cycles that lead to more complex thermochemical treatments.

Note: Even though aluminum melts at *ca*. 660 °C, heating up to 600 °C damages the aluminum envelope and allows combustion of SFH to proceed.

### Procedure

• Place the aluminum envelope containing SFH in the oven.
• Initiate the heating program.

Caution: care should be taken that the metallic envelope does not short circuit the heating elements in the oven.

Note: heating at 10 °C min^−1^ up to *T*_final_ °C (*T*_final_ 150–500 °C, every 50 °C) and leaving 2 h at *T*_final_ proved to be suitable in all 48 independent experiments that I performed.

• Immediately after the thermal treatment finishes, remove the aluminum envelope from the oven.

Caution: before removing the envelope, turn the oven off; then, use a crucible tong while wearing goggles and isolating gloves. Though thermally treated aluminum envelopes cool fast, they may burn if handled carelessly.

Step 3: Open the envelope to obtain the carbonized SFH.

### Materials

• Thermally treated aluminum envelope.
• Scissors.

### Procedure

• Gently hit the envelope against the bench to allow the solid inside to fall inside the envelope.
• Cut out the upper side of the envelope with scissors.

Tip: work in a fume hood or outdoors as smelly, gaseous products forms during the thermal treatment.

• Transfer the solid product to a flask where it will be stored.

## Additional information

**Fig. 1:**
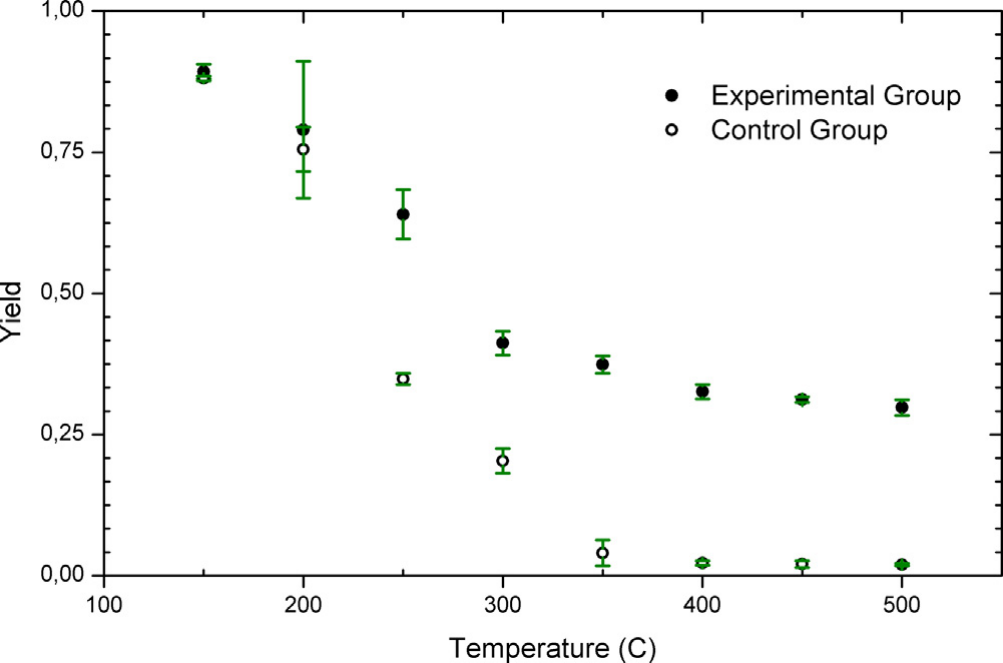
Mean values of yields (●) obtained when thermally treating SFH within airtight envelopes made with aluminum foil (experimental group) and mean values of yields (○) obtained when SFH placed in an open box made with aluminum foil (control group). Samples were heated at 10 °C min^−1^ up to the final temperature, where the sample remained isothermally 2 h. Three independent experiments were performed at each experimental condition. Yields were calculated as *m_f_*/*m*_*0*,_ where *m_f_* represents the mass of SFH before and *m*_0_ after the thermal treatment. All results were expressed as $\bar{x}$± 3δ (see Appendix A).

Three complementary indicators of a successful carbonization of biomass are yield of the process (see definition in caption of Fig. 1), elemental composition and appearance of the solid product.

Thermochemical processes converting biomass into charcoal typically have yields somewhat lower than 0.50. Normally, SFH consists of 42–50% C, 35–43% O, 5–6% H and 1% N [4]. Ideally, carbonization of SFH could have yields of 0.43–0.50 if all elements but C would be removed. However, in practice, some C originally present in SFH forms volatile products, while some O, H and N remain in the solid product. In case that oxygen reaches the solid during carbonization, yields would dramatically drop. In case of complete combustion of the organic components of SFH, yields around 0.01–0.05 should be expected due to formation of ashes. Values of yields obtained following our method (see Fig. 1) are consistent with values obtained when carbonizing SFH with other methods [5], [6], [7].

Yields obtained with SFH within envelopes made with aluminum significantly differed from the yields obtained when heating SFH in open boxes made with aluminum foil in the temperature range 250–500 °C. Open boxes allow O_2_ to reach the surface of the solid formed during the thermochemical process. Thus, the solid reacts with O_2_ and forms volatile products. Yields in the temperature range 400–500 °C showed a constant value of 2.1 ± 0.8%, indicating that complete combustion of organic mater already occurred. The experiments performed with open boxes constituted our control group, while the experiments performed with airtight envelopes our experimental group.

**Fig. 2:**
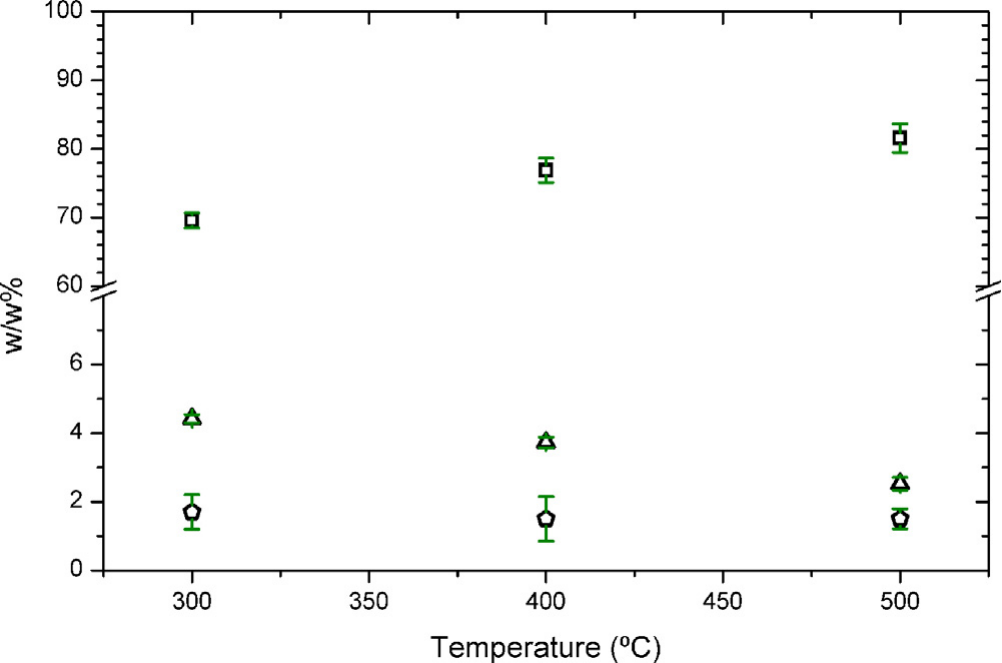
Elemental composition of the solid product obtained after the thermal treatment of SFH within the metallic envelops (experimental group) in terms of carbon (square, top), hydrogen (triangle, middle) and nitrogen (pentagon, bottom). Samples were heated at 10 °C min^−1^ up to the final temperature, where the sample remained isothermally 2 h. Three independent experiments were performed at each experimental condition. All results were expressed as $\bar{x}$± 3δ (see Appendix A).

Another indicator of a successful carbonization is the elemental composition of the solid product in terms of C, H and N, which should show a solid richer in C and poorer in all other elements than the starting material. The thermochemical treatment of SFH with aluminum envelopes showed the expected tendency (see Fig. 2). The solid product enriched in C as the maximum temperature of the treatment rose. Simultaneously, the solid experienced a considerable reduction in H, while the N content remained constant. Elemental compositions in solid products obtained following our method are consistent with values obtained when carbonizing SFH with other methods [8].

A third indicator of a successful carbonization is the appearance of the solid product. When thermally treated in absence of air, SFH show a typical change in pattern, shape and texture. The typical white-and-black, stripped pattern of SFH starts darkening at 250 °C and blackens at *ca*. 300 °C [9], [10]. Furthermore, SFH anisotropically contracts during carbonization forming a solid product that resembles the shape of its precursor [10]. Last, the solid product can be handled without loosing its shape. Contrastingly, when thermally treated in presence of air, appearance of SFH changes differently. The stripped pattern of SFH darkens already at 200 °C and keeps so up to 300 °C. Above 300 °C, a light gray powder forms.

## Appendix A

### Calculating mean values and uncertainties

I calculated mean values of yields and of elemental compositions with Eq. (C.1), uncertainties in individual values of yields with Eq. (C.2), and uncertainties in mean values of yields and elemental composition with Eq. (C.3) (see [11] p. 60). I considered uncertainties in *m_f_* (mass of solid after thermal treatment) and *m_i_* (mass of biomass) as well as *x_i_* to be independent and random.

(C.1) $$\bar{x}=\frac{\sum_{i=3}^{n}x_{i}}{3}$$

(C.2) $$\frac{\text{δ}y}{\left|y\right|}=\sqrt{\left({\left(\frac{\text{δ}m_{f}}{m_{f}}\right)}^{2}+{\left(\frac{\text{δ}m_{i}}{m_{i}}\right)}^{2}\right)}$$

(C.3) $$\delta x=\sqrt{\sum_{i=1}^{3}{\left({\delta x}_{i}\right)}^{2}}$$
